# Gastrointestinal Manifestations As the Initial Presentation of Neurological Disease

**DOI:** 10.7759/cureus.93615

**Published:** 2025-09-30

**Authors:** Pravarshini Datla, Sowmya Gopalan, Preetam Arthur

**Affiliations:** 1 Internal Medicine, Sri Ramachandra Institute of Higher Education and Research, Chennai, IND

**Keywords:** case report, cataract, diabetes, diarrhoea, electromyography, gastrointestinal manifestations, myotonic discharges, myotonic dystrophy, percussion myotonia, young adult

## Abstract

Myotonic dystrophy (MD) is a multi-system autosomal dominant disorder characterised by progressive muscular weakness, with ocular, cardiac, and endocrine involvement. Gastrointestinal manifestations are not a classical feature of myotonic dystrophy and are hence often misdiagnosed if they precede musculoskeletal manifestations. We present a rare case of chronic diarrhoea as the initial manifestation of myotonic dystrophy in a young adult male with a history of diabetes mellitus and early onset cataract.

## Introduction

Myotonic dystrophy (MD) is a multisystem, autosomal dominant disorder involving neuromuscular, ocular, and endocrine systems [1,2]. There are two main types: type I, called Steinert disease, and a milder type, type II [3]. Gastrointestinal (GI) symptoms are frequently under-recognised and under-reported, and can precede classical musculoskeletal features, leading to delayed diagnosis [4]. GI manifestations in MD affect the entire digestive tract, including symptoms like dysphagia and reflux, which are the most common, abdominal pain, constipation, and, less commonly, diarrhoea [5,6]. This report presents a rare case of chronic diarrhoea as the initial GI manifestation of MD, emphasising diagnostic challenges and the need for raised clinical suspicion. This case highlights the importance of considering MD in young adults with unexplained GI symptoms.

## Case presentation

A 23-year-old male with a history of type 2 diabetes mellitus on insulin therapy for five years and left cataract surgery three years back presented with complaints of loose stools for three months. The patient had three to four episodes of loose stools per day, which were watery, yellow in colour, non-blood-stained, and non-mucoid. The patient also had a history of weakness for two months, which slowly increased with time. There was no history of fever, abdominal pain, vomiting, hematochezia, or recent travel. The patient had no family history of similar complaints, diabetes, or muscle disorders. The patient had multiple outpatient visits in other clinics previously and had been treated symptomatically with no clinical improvement. 

Diabetic peripheral and autonomic neuropathy were considered as a differential diagnosis during the current admission. The patient had no heart rate variability, postural hypotension, or bowel and bladder incontinence.

**Figure 1:**
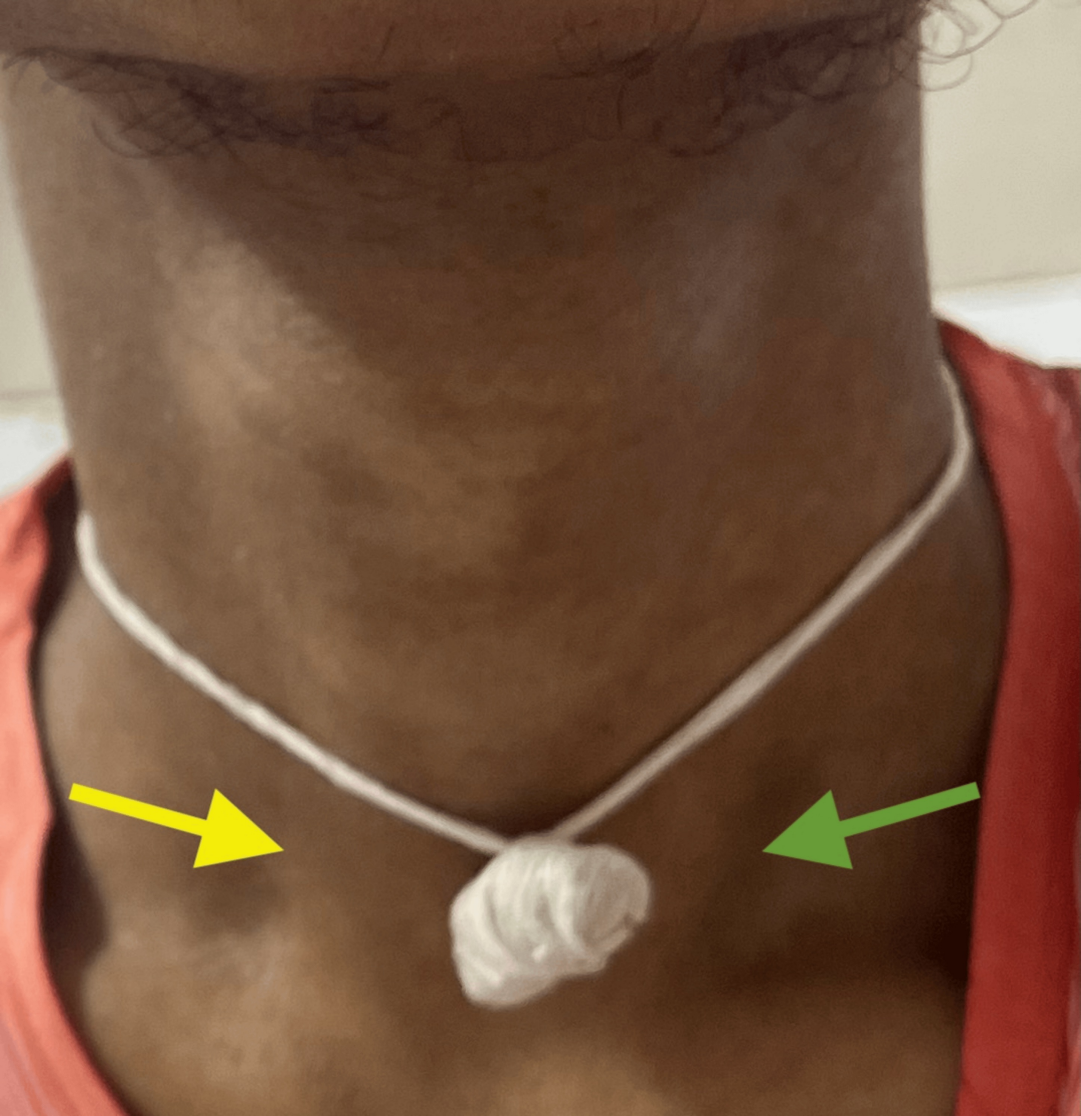
Wasting of right (yellow arrow) and left (green arrow) sternocleidomastoid muscles

Physical examination revealed a thin, asthenic male (body mass index: 18 kg/m²), with myopathic facies, temporal wasting, and early frontal balding. There was wasting of bilateral sternocleidomastoid muscles as seen in Figure 1. 

**Figure 2:**
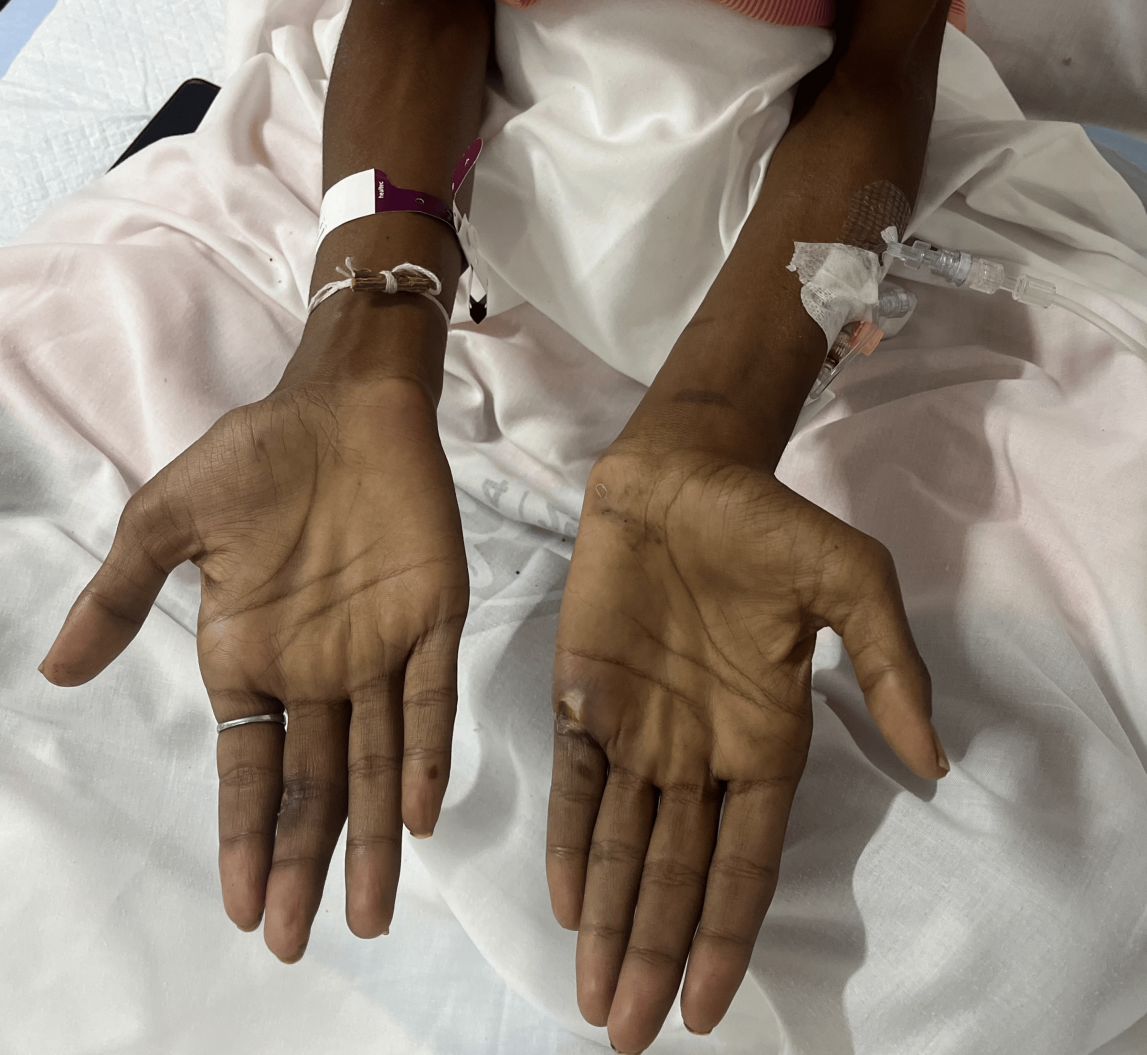
Severe wasting (thenar, hypothenar and interossei) of the upper limbs in the patient

**Figure 3:**
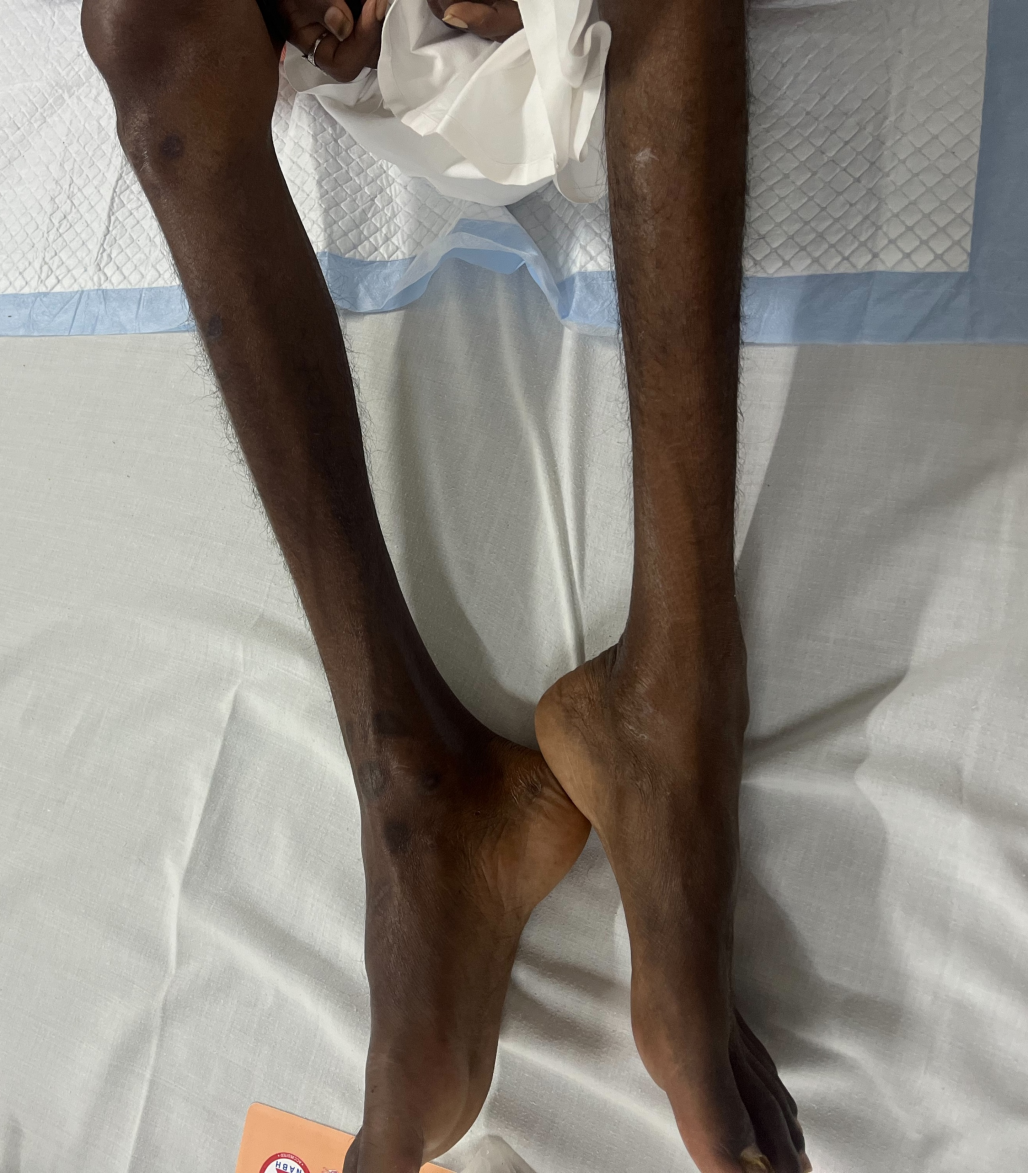
Severe wasting of bilateral lower limbs in the patient

There was percussion myotonia seen in the hand muscles. Neurological assessment revealed distal muscle (thenar, hypothenar, and interossei) wasting in both upper limbs (Figure 2), along with distal muscle wasting of lower limbs (Figure 3), as well as weakness with a power of 3/5 in the lower limb muscles and 4/5 in the upper limb muscles, with preserved deep tendon reflexes. The abdomen was soft with no organomegaly or tenderness. 

Basic laboratory investigations were normal. Serum C-peptide was increased to 4ng/ml. Serum glycated haemoglobin (HbA1c) was 14% and serum testosterone was decreased to 1.69ng/ml (Table 1). The electrocardiograph was normal.

**Table 1:** Basic laboratory investigations BUN - blood urea nitrogen; HbA1c - glycated hemoglobin; FBS - fasting blood sugar; PPBS - post prandial blood sugar; FT4 - free thyroxine; TSH - thyroid stimulating hormone

Test	Value	Reference value
Hemoglobin	13 g/dL	12-17 g/dL
Total count	5600 cumm	4000-11000 cumm
Platelet count	2.2 lakhs/ cumm	1.5-4.5 laksh/cumm
BUN	11 mg/dL	7.9-20.1 mg/dL
Creatinine	0.8 mg/dL	0.8-1.3 mg/dL
HbA1c	14%	<5.6%
FBS	198 mg/dL	70-110 mg/dL
PPBS	266 mg/dL	80-140 mg/dL
Testosterone	1.69 ng/mL	1.75-7.81 ng/mL
Cortisol	20 µg/dL	5-23 µg/dL
FT4	1.72 ng/dL	0.8 to 1.8 ng/dL
TSH	2.47 µIU/mL	0.35- 5.5 µIU/mL

**Figure 4:**
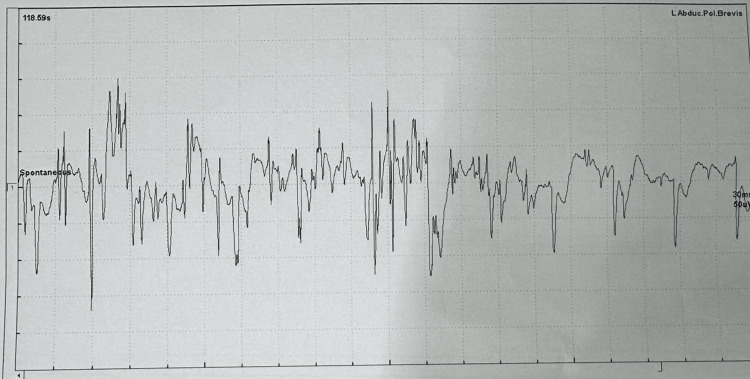
Electromyography showing myotonic discharges in the left abductor pollicis brevis

**Figure 5:**
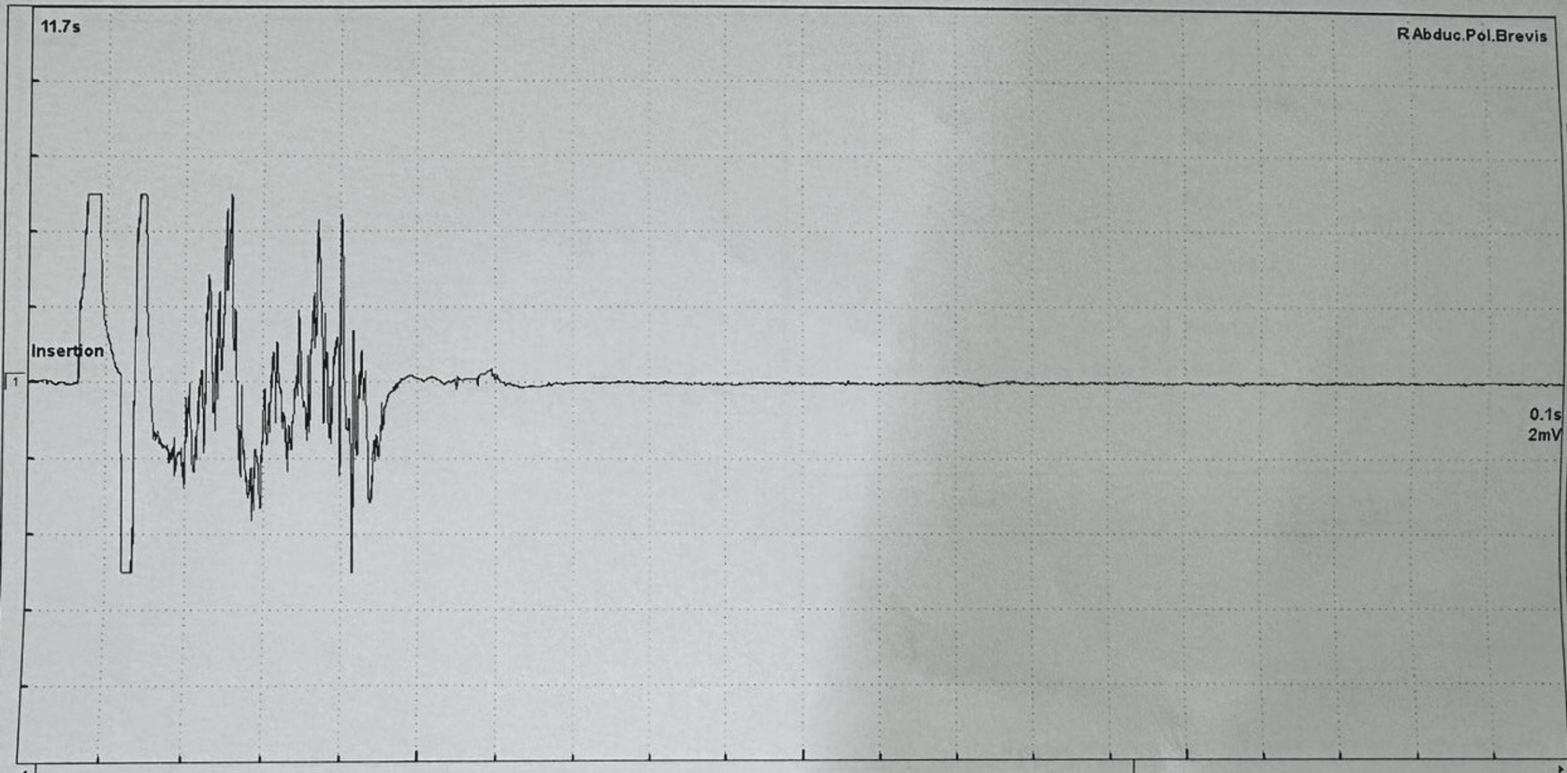
Electromyography showing myotonic discharges in the right abductor pollicis brevis

Further evaluation for GI symptoms included stool analysis, which was negative for pathogens, ova, cysts, and fat. Computed tomography of the abdomen was normal. Colonoscopy was normal. A breath test for small intestinal bacterial overgrowth was not done due to financial constraints. Thyroid function was normal. Nerve conduction studies revealed reduced compound muscle action potential amplitudes in the right median, ulnar, and bilateral tibial nerves, with normal distal latencies and conduction velocities. The amplitudes of the bilateral peroneal nerves were absent. This was suggestive of a myopathic pattern. Hence, Electromyography was performed, which revealed a classic myogenic pattern in the tested muscles, with myogenic discharges in the left (Figure 4) and right (Figure 5) abductor pollicis brevis, confirming the diagnosis of myotonic dystrophy along with other multisystem involvement. Genetic testing was not done due to financial constraints. 

The patient was continued on basal-bolus insulin therapy according to blood glucose levels. He was treated with norfloxacin and cholestyramine and had resolution of lower gastrointestinal symptoms. The patient was also started on phenytoin and is on follow-up. 

## Discussion

Myotonic dystrophy is a neurological disease with myotonic phenomena and progressive muscular weakness [1]. It is a multi-system disorder and can cause abnormalities in cardiac conduction, cataract, endocrine disorders, and sexual and reproductive disorders [2]. Endocrinopathies in the form of diabetes mellitus, thyroid dysfunction, and hypogonadism are predominant [3].

In myotonic dystrophy, GI involvement may affect the entire digestive tract, with dysphagia and constipation reported as the most frequent symptoms [4,5]. Diarrhoea is less frequent but can occur due to reduced peristalsis or gastric dysmotility along with bacterial overgrowth [6,7]. Exocrine pancreatic insufficiency as a causative mechanism for diarrhoea is also being studied, but concrete evidence is not available yet [8]. There is only a mild correlation between gastrointestinal disturbances and severity of muscle damage; however, there is a strong correlation between the severity of gastrointestinal manifestations and the duration of myotonic dystrophy [9,10]. In some patients with diarrhoea, radiology revealed dilatation of the small intestine with decreased GI motility and delay in barium transit [11]. Symptomatic treatment of diarrhoea with norfloxacin, either alone or in combination with cholestyramine, has been proven to be useful in patients [12]. Sodium channel blockers like mexiletine and phenytoin have been used for reducing the delayed muscle relaxation associated with myotonia as they raise the depolarisation threshold of the muscle membranes [6].

This case highlights the diagnostic challenge in patients with overlapping endocrine, ocular, and neuromuscular disorders. The presence of early cataracts, myopathic facies, and diabetes should prompt consideration of myotonic dystrophy in young adults presenting with unexplained gastrointestinal symptoms. Early diagnosis enables optimal symptomatic management and genetic counselling.

## Conclusions

Gastrointestinal dysmotility may be underrecognized in myotonic dystrophy, can precede neuromuscular involvement, and significantly impact quality of life. Early recognition can alleviate symptoms, improve quality of life, and also aid in genetic counselling and family screening.
